# Primary nodal anthracosis identified by EBUS-TBNA as a cause of FDG PET/CT positive mediastinal lymphadenopathy

**DOI:** 10.1016/j.rmcr.2013.09.005

**Published:** 2013-09-27

**Authors:** Richard J. Hewitt, Corrina Wright, David Adeboyeku, Dan Ornadel, Matthew Berry, Melissa Wickremasinghe, Andrew Wright, Annemarie Sykes, Onn Min Kon

**Affiliations:** a Chest and Allergy Department, St. Mary's Hospital, Imperial College Healthcare NHS Trust, Praed Street, London W2 1NY, UK; b Cytopathology, St. Mary's Hospital, Imperial College Healthcare NHS Trust, Praed Street, London W2 1NY, UK; c Respiratory Medicine, Central Middlesex Hospital, The North West London Hospitals NHS Trust, Acton Lane, London NW10 7NS, UK; d Respiratory Medicine, Northwick Park Hospital, The North West London Hospitals NHS Trust, Watford Road, Harrow, Middlesex HA1 3UJ, UK; e Radiology, St. Mary's Hospital, Imperial College Healthcare NHS Trust, Praed Street, London W2 1NY, UK; f Imperial College London, St Mary's Campus, Norfolk Place, London W2 1PG, UK

**Keywords:** Anthracosis, EBUS-TBNA, FDG PET/CT, Mediastinal lymphapenopathy

## Abstract

Isolated mediastinal lymphadenopathy can result from a number of potentially serious aetiologies. Traditionally those presenting with mediastinal lymphadenopathy would undergo mediastinoscopy to elucidate a final diagnosis or receive empirical treatment. There is now increased utilization of endobronchial ultrasound-guided transbronchial needle aspiration (EBUS-TBNA), in this setting.

Five cases of mediastinal lymphadenopathy are presented here in which lymph node anthracosis was identified as the primary diagnosis using EBUS-TBNA. They were female, non-smokers presenting with non-specific symptoms, who retrospectively reported cooking over wood fires. Four were from South Asia. Three were investigated by F-18-fluorodeoxyglucose positron emission tomography/computed tomography (FDG PET/CT) scanning and increased signal was identified in the anthracotic nodes sampled.

With expansion of PET/CT and EBUS-TBNA services it is likely that primary nodal anthracosis will be encountered more frequently and should be considered in the differential diagnosis of those with PET/CT positive lymphadenopathy. It may mimic pathologies including tuberculosis and malignancy, thus accurate sampling and follow-up are essential.

## Abbreviations list

CT: computed tomography
EBUS-TBNA: endobronchial ultrasound-guided transbronchial needle aspiration
FDG PET/CT: F-18-fluorodeoxyglucose positron emission tomography/computed tomography
PCR: polymerase chain reaction
SUV: standardised uptake value
TB: tuberculosis
VATS: video assisted thoracoscopic surgery

## 1 Introduction

Enlarged mediastinal lymph nodes can result from a number of potentially serious aetiologies including tuberculosis (TB), carcinoma, lymphoma, sarcoidosis or can be benign. Investigation traditionally involved mediastinoscopy but this has been mainly superseded by endobronchial ultrasound-guided transbronchial needle aspiration (EBUS-TBNA). This procedure is less invasive and can sample an increased range of lymph nodes [1]. EBUS-TBNA has been demonstrated to be a valuable diagnostic tool in lung cancer [2], sarcoidosis [3] and tuberculosis [4].

The accumulation of a black, carbon-containing pigment, within the airways or lungs of those exposed to coal dust, biomass smoke or air pollution is well recognized [5], [6]. Anthracosis has also been described in mediastinal nodes mimicking TB [7] and malignancy [8], [9]. Invasive thoracoscopy or mediastinoscopy was required to elucidate anthracosis as the final diagnosis in these cases [7], [9]. Anthracosis has also been identified by transoesophageal endosonography with fine needle aspiration in a case of anthracosis presenting on F-18-fluorodeoxyglucose positron emission tomography/computed tomography (FDG-PET/CT) with hypermetabolic mediastinal lymphadenopathy mimicking malignancy [8]. With increasing use of EBUS-TBNA in the investigation of mediastinal lymphadenopathy, anthracosis may be identified more frequently.

## 2 Methods

The cases described in this report are from a regional EBUS centre in North West London. This centre receives referrals from outlying hospitals to perform both diagnostic and staging EBUS examinations.

This was a retrospective case series driven by the clinical observation that there appeared to be cases of PET avid lymph nodes that were eventually proven to only have anthracotic changes. An audit of the case load from January to June 2012 identified cases where no other diagnosis was finally made apart from anthracotic change within lymph nodes.

The decision to utilise a PET scan prior to EBUS was made on standard clinical grounds by the requesting respiratory physician as the initial presumptive diagnosis would have been that of probable malignancy. PET is used as a staging tool and to guide the requirement for further sampling. Similarly EBUS-TBNA is used in this region as the initial sampling modality for suitable lymph node stations and would have been requested at the discretion of the local referring multi-disciplinary teams.

Mycobacterial cultures were routinely sent in all cases given the presence of mediastinal lymphadenopathy. Other bacterial or fungal cultures were only sent if there were coincidental parenchymal abnormalities; in this series this was irrelevant in all but Case 2.

Having identified cases where the rapid cytological evaluation defined only anthracosis, the clinician involved took a more detailed exposure history directly from the patient with a particular focus on whether biomass fuel exposure was a factor.

## 3 Case reports

### 3.1 Case 1

A 67-year old Afghani woman was referred after an incidental finding of right hilar and paratracheal lymphadenopathy during investigations for left-sided chest pain. She reported breathlessness on climbing stairs. Past medical history included type 2 diabetes mellitus, hypertension and TB fully treated in Afghanistan 35 years previously. She was a lifelong non-smoker. Examination was unremarkable.

**Fig. 1:**
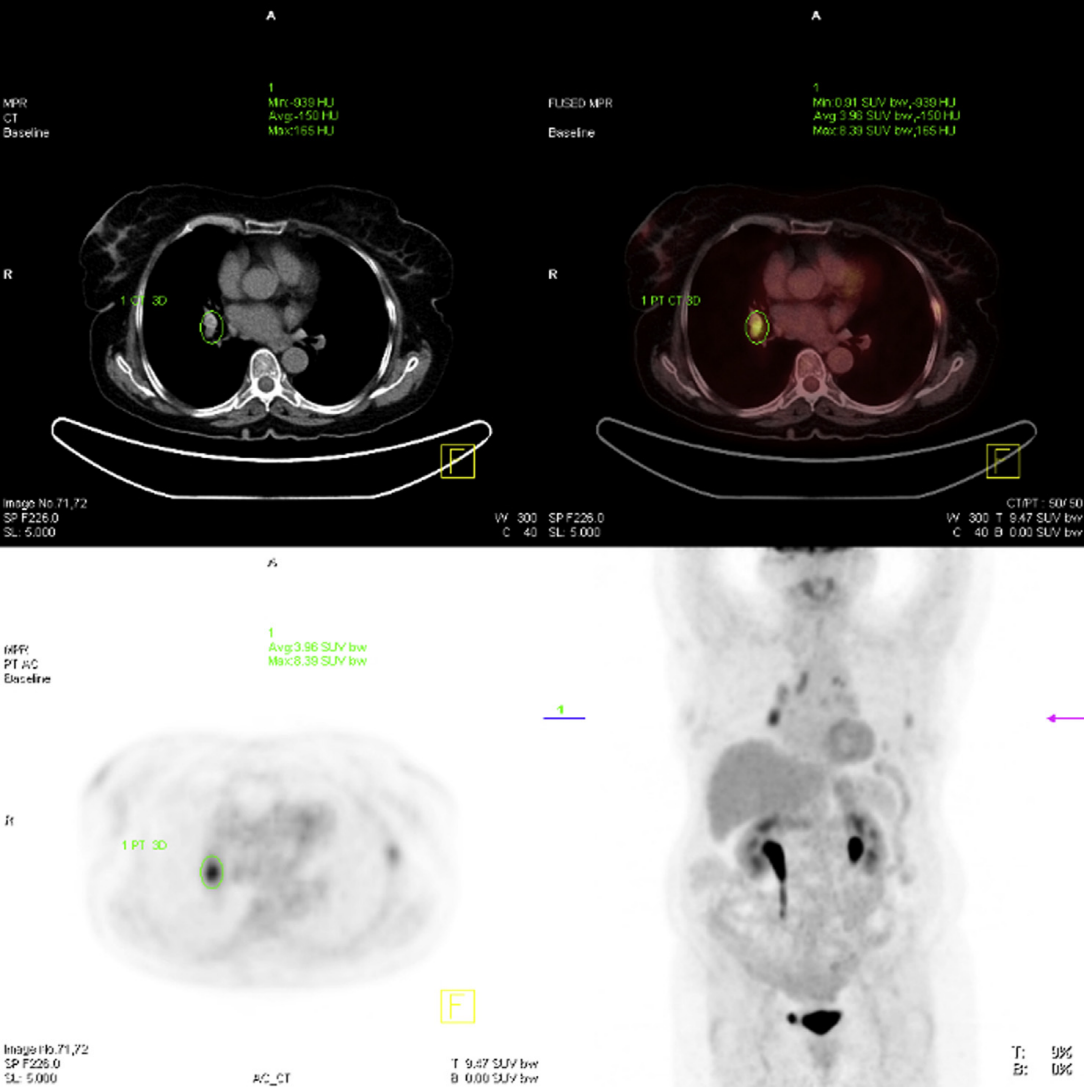
A FDG PET/CT scan demonstrated high-grade metabolic activity in the right hilar soft tissue lesion, maximum SUV 8.4, measuring approximately 2.4 cm. There are metabolically active right hilar, paratracheal, and prevascular lymphadenopathy.

**Fig. 2:**
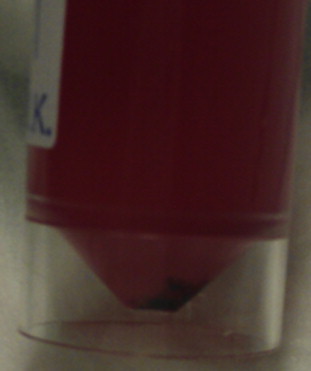
Macroscopic appearance; Black pigment obtained on EBUS-TBNA of subcarinal, right lower paratracheal lymph nodes (Station 4R; as defined by the International Association for the Study of Lung Cancer lymph node map [11]) and right hilar mediastinal lymph nodes.

**Fig. 3:**
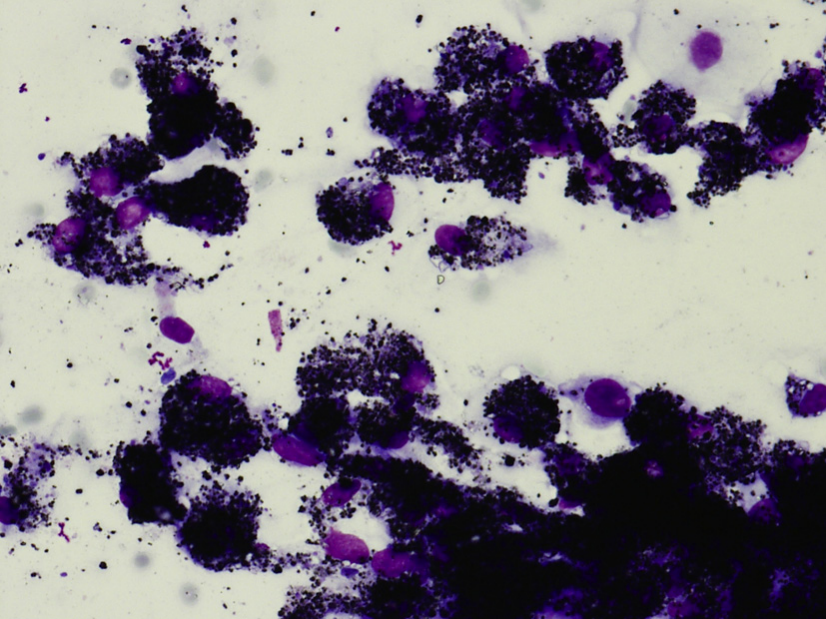
Cytological findings on EBUS-TBNA of the right lower paratracheal lymph nodes (Station 4R) [11]. May-Grunwald Giemsa stained direct smear preparations. Abundant anthracotic pigment in the cytoplasm of macrophages (×400, original magnification).

A T-Spot test was positive, consistent with her previous TB, but TBNA samples were auramine, culture and polymerase chain reaction (PCR) negative for TB. A computed tomography (CT) scan performed during inpatient investigations identified a left rib fracture in addition to incidental right-sided hilar and paratracheal lymphadenopathy. An FDG-PET scan demonstrated increased metabolic activity in the right paratracheal node with a maximum standardised uptake value (SUV) of 8.4 (normal values <2.7) [10] (Fig. 1). On EBUS-TBNA of subcarinal, paratracheal and right hilar mediastinal lymph nodes, black pigment was obtained macroscopically (Fig. 2). On microscopic examination the aspirate was abundantly cellular with a population of anthracotic macrophages that were both singly dispersed and in variously sized aggregates (Fig. 3). There was a single foreign body type, multinucleated giant cell adjacent to one aggregate of anthracotic macrophages. No necrosis or malignant cells were seen. She retrospectively reported cooking over open fires.

A follow-up CT scan at 6-months showed completely stable appearances, with some pulmonary nodules, likely intrapulmonary lymph nodes, and part calcified mediastinal lymphadenopathy. Clinically she remained well having gained 2 kg in weight, with no night sweats, fevers, cough or breathlessness. She is due further follow-up at 18 months.

### 3.2 Case 2

A 74-year-old woman with a 20-year history of non-productive cough underwent a CT scan as part of evaluation of a left lower lobe calcified nodule. She was otherwise healthy and examination was unremarkable. The CT scan identified curvilinear shadowing in the right middle lobe and a second non-calcified nodule inferiorly in the left lower lobe. Right middle lobe bronchial washings were culture negative for bacterial or fungal infection and there were no malignant cells. Auramine stain and TB cultures were negative.

An FDG-PET scan demonstrated normal metabolic activity in the nodules but identified increased activity in enlarged right paratracheal, para-oesophageal and bilateral hilar lymph nodes (SUV 5-7) and an incidental right adrenal adenoma. EBUS-TBNA of the right paratracheal lymph node macroscopically showed lightly bloodstained material that contained small black flecks up to two millimetres in diameter. Microscopically there were striking amounts of anthracotic macrophages, arranged in aggregates and as singly dispersed cells. No multinucleated giant cells, necrosis or malignant cells were seen and was auramine and culture negative for TB.

A follow-up CT scan at 12 months showed no significant change in the appearance of the lung with no new nodules or significant change in the existing nodules. She was asymptomatic and due further follow-up at 18 months.

### 3.3 Case 3

A 68-year old Pakistani woman presented with persistent cough and wheeze. Past medical history included atrial fibrillation, hypertension, hypothyroidism, asthma and IgA nephropathy. Examination was unremarkable. CT chest demonstrated mediastinal and hilar lymphadenopathy, and a small, non-specific left-sided pulmonary nodule.

An EBUS-TBNA was performed on the mediastinal and hilar lymph nodes. No black pigment was seen macroscopically. Microscopically large numbers of anthracotic macrophages were seen, singly distributed and in dense clusters. There was no multinucleated giant cell reaction, necrosis or malignant cells and was culture, smear and PCR negative for TB.

A follow-up CT scan at 9 months revealed unchanged appearances of the pulmonary nodule and mediastinal lymphadenopathy. She remained clinically well and is due a further follow-up CT imaging at 24 months.

### 3.4 Case 4

A 65-year old Punjabi woman with a 3-year history of cough was referred for investigation. She denied any associated symptoms. Ten months prior to presentation she had been treated for presumed TB, on the basis of enlarged mediastinal lymph nodes on CT imaging and a strongly positive mantoux test. She was fully compliant with anti-tuberculous medication. She had hypertension and had been treated for cervical carcinoma aged 28. She moved to the UK in her teens and had never smoked. Examination was unremarkable.

A CT scan performed 6 months after completion of TB treatment revealed persistence of right paratracheal, subcarinal, right hilar and aortopulmonary lymhadenopathy and partial atelectasis of the postero-lateral segment of the right middle lobe. EBUS-TBNA was performed on the right paratracheal and subcarinal lymph nodes. This was highly cellular black pigmented material microscopically with anthracotic macrophages in collections and as single dispersed cells. No multinucleated giant cells, necrosis or malignant cells were seen. Culture, smear and PCR were negative for TB.

At follow-up she was well with resolution of her cough. She is due follow-up lung function testing at 12 months.

### 3.5 Case 5

A 73-year old Afghani woman presented with 3 weeks of productive cough and an 8-month history of hoarse voice and coughing whilst eating. She denied weight loss, fever or night sweats. She was a lifelong non-smoker and had a past medical history of hypertension and type 2 diabetes mellitus. Her father had TB aged 35. Examination identified a firm, enlarged thyroid, a fixed monophonic wheeze in left mid zone and a left vocal cord paralysis. She was biochemically euthyroid.

A CT scan identified mediastinal and left hilar lymphadenopathy, abnormal soft tissue surrounding the left main bronchus, multiple bilateral calcified pulmonary nodules and a multi-nodular goitre. PET/CT scan demonstrated activity in the mediastinal lymph nodes with SUV of 8.4.

Bronchoscopy identified an endobronchial soft tissue mass but endobronchial biopsies and washings failed to identify malignant cells or granuloma, demonstrating only inflammation and squamous metaplasia. Bronchial washings were auramine, culture and PCR negative for TB. EBUS-TBNA of the mediastinal mass showed black material macroscopically. On microscopy there were abundant anthracotic macrophages which were distributed singly and in aggregates. No multinucleated giant cells, necrosis or malignant cells were seen. On further questioning she admitted cooking on wood fires in Afghanistan, and remembered inhaling dust and sand during dust storms.

The patient declined further investigation with repeated EBUS or video assisted thoracoscopy surgery (VATS), preferring a period of symptomatic and radiological observation.

A follow-up CT scan showed no change in the size of mediastinal nodes at 10 months. Despite continuing to suffer a left vocal cord palsy secondary to aortopulmonary lymphadenopathy, she remains well at 18 months with no other aetiology found.

## 4 Discussion

This report describes five cases of mediastinal lymphadenopathy in which lymph node anthracosis was identified as the final primary diagnosis using EBUS-TBNA. They were female non-smokers who retrospectively reported cooking over wood fires. Four cases were from South Asia. Extended exposure to biomass smoke has previously been associated with domestically acquired particulate lung disease in non-smoking, Iranian women [12], [13], however mediastinal lymphadenopathy was not a feature of these cases and anthracotic plaques were found in the airways [13].

Extrapulmonary anthracosis is rare, but can present as mediastinal lymphadenopathy mimicking tuberculosis [7] and malignancy [8], [9]. In the pre-EBUS era mediastinoscopy or transeosophageal endosonography with fine needle aspirate were methods required for diagnosis but can be associated with significant morbidity [1], [14], [15] and is only available in cardiothoracic centers. It is possible that pre-EBUS, cases of mediastinal lymphadenopathy in elderly, Asian, non-smokers would have been treated empirically for TB rather than further investigated, as in Case 4. EBUS-TBNA has demonstrated a diagnostic sensitivity of 94% in patients with intra-thoracic tuberculous lymphadenopathy [4] and combined with PCR techniques has allowed rapid identification and targeted treatment of drug-resistant strains [16]. With increasing utilization of EBUS-TBNA for this purpose, alternative diagnoses may be more frequently identified. All cases documented in our series were TB smear, culture and PCR negative, none have developed TB or malignancy on follow-up.

Three of our cases were investigated by FDG-PET/CT scanning and increased signal was identified in the anthracotic nodes sampled. Anthracotic lymph nodes in the neck [17], hilar region and mediastinum [8] and anthracotic pulmonary nodules [18] have all been reported to display increased FDG uptake on PET scanning. Inflammatory processes are responsible for false positive lymph node status on PET/CT evaluation of early stage non-small cell lung carcinoma, with anthracosis noted as a potential cause [9].

On the basis of the PET/CT imaging and histopathological findings of granuloma-like aggregates of anthracotic macrophages, parallels can be drawn with conditions such as sarcoidosis [19]. Increased FDG uptake has been shown in the mediastinal lymph nodes of patients with sarcoidosis [20]. Anthracotic material could provide persistent antigenic stimulation to macrophages with resultant inflammatory activity highlighted on PET/CT.

Dust-laden alveolar macrophages have been shown to migrate to mediastinal lymph nodes following inhalation exposure to inorganic dust fibers in animals [21]. This process of clearance may also operate in anthracosis.

Importantly, anthracosis presenting as PET/CT positive lymphadenopathy can coexist or mimic other conditions including tuberculosis [8], and non-small cell lung cancer [22]. This exemplifies the importance of rigorous clinical and radiological follow-up with the aim of identifying any co-existing or underlying pathologies. All cases described in this series were followed-up and remained well at 12 months. Radiologically, the lung and mediastinal appearances remained stable in those that had a repeat CT scan.

In summary we present five cases of mediastinal lymphadenopathy associated with anthracosis and exposure to wood smoke in South Asian women. The nodes were metabolically active on PET/CT and radiologically indistinguishable from those in malignancy, TB or sarcoidosis. EBUS-TBNA enabled a diagnosis of primary nodal anthracosis over other possible aetiologies, and avoidance of unnecessary empirical treatment or further investigations. Accurate lymph node sampling with EBUS-TBNA to obtain a diagnosis and regular follow-up are key aspects of management. Primary nodal anthracosis should be considered in the differential diagnosis of FDG PET/CT positive mediastinal lymphadenopathy and respiratory physicians should be inquisitive about domestic wood smoke exposure.

## Conflicts of interest

None.
